# OLDER ADULT DISASTER RECOVERY FOLLOWING HURRICANE KATRINA

**DOI:** 10.1093/geroni/igz038.2076

**Published:** 2019-11-08

**Authors:** Alexis Merdjanoff, David Abramson, Rachael Piltch-Loeb, Yoon Soo Park

**Affiliations:** 1 New York University, New York, New York, United States; 2 University of Illinois - Chicago, Chicago, Illinois, United States

## Abstract

This study explores the effects of environmental disruption on older adult well-being, recovery and resilience following Hurricane Katrina. It is based upon the Gulf Coast Child and Family Health Study, a longitudinal cohort of 1,079 residents from Louisiana and Mississippi highly affected by the storm. Using five waves of data collected over the last 12 years, analyses examine the drivers of long-term recovery by age group, including factors such as household income, physical health, mental health, stable housing and social support. Path analyses compare the influence of these drivers on recovery among younger adults (18-54), the young-old (55-64), mid-old (65-74) and old-old (75+). Results demonstrate that each age group relies on specific factors to improve their recovery, and that only a small number of factors are critical for older adult recovery. Results can help identify points of intervention for disaster recovery planning that can facilitate long-term recovery for older adults.

